# Dopamine Burden Induced the Inactivation of Sonic Hedgehog Signaling to Cognitive Decline in Minimal Hepatic Encephalopathy

**DOI:** 10.14336/AD.2016.1123

**Published:** 2017-07-21

**Authors:** Saidan Ding, Jianjing Yang, Xueli Huang, Leping Liu, Jiangnan Hu, Zhu Xu, Qichuan Zhuge

**Affiliations:** ^1^Zhejiang Provincial Key Laboratory of Aging and Neurological Disease Research, Department of Surgery Laboratory, the First Affiliated Hospital, Wenzhou Medical University, Zhejiang 325000, China; ^2^Department of Neurosurgery, the First Affiliated Hospital, Wenzhou Medical University, Zhejiang 325000, China; ^3^School of Pharmaceutical Sciences, Wenzhou Medical University, Zhejiang 325000, China; ^4^Institute for Healthy Aging, University of North Texas Health Science Center, Fort Worth, Texas 76107, USA

**Keywords:** minimal hepatic encephalopathy, naringin, dopamine, sonic hedgehog pathway, neurotrophin

## Abstract

Minimal hepatic encephalopathy (MHE) is induced by elevated intracranial dopamine (DA). The relationship of the Shh pathway with memory loss in MHE, however, is elusive. In the current study, rats with MHE induced with DA displayed downregulation of the Shh pathway. Additionally, injection of Shh into MHE/DA-treated rats reversed downregulation of BDNF/NT3, whereas administration of cyclopamine (Cyc) enhanced the inhibition of expression of BDNF/NT3. Furthermore, naringin (Nrg) substantially prevented cognitive impairment in MHE/DA-treated rats and upregulated the Shh pathway, paralleling the elevated expression of BDNF/NT3. Overall, our results indicate that the Shh pathway can induce the expression of BDNF/NT3, and DA causes memory loss by inactivation of Shh pathway signaling to BDNF/NT3 in MHE rats, which is reversed by Nrg. Our study may provide new theory basis of pathogenesis and therapeutic target of MHE.

Minimal hepatic encephalopathy (MHE), the mildest form of hepatic encephalopathy (HE)[1], refers to the absence of clinical evidence of hepatic encephalopathy and subtle changes in cognitive function observable by electrophysiological parameters [2]. Our previous study found that the pathogenesis of MHE may be associated with elevated dopamine (DA) in cirrhotic liver: the DA level was elevated in cirrhotic liver, crossed the blood-brain barrier, went into the brain of MHE rats, and inhibited learning and memory by blocking the glutamate-nitric oxide-cyclic guanosine monophosphate pathway [3]. However, the pathophysiological mechanisms by which DA leads to MHE are not clearly understood.

Sonic hedgehog (Shh) has been the most studied member of the hedgehog (Hh) signaling pathway in vertebrates [4]. A transmembrane receptor, patched 1 (Ptch1), has been identified as the receptor for processed Hh ligands. In the absence of ligand, Ptch1 represses the activity of Smoothened (Smo). On ligand binding, the repression of Smo is alleviated, and Smo initiates a signaling cascade that results in the translocation of Gli transcription factors into the nucleus [5]. Shh was reported to regulate synaptic plasticity by a mechanism involving both pre- and post-synaptic processes [6, 7]. It was hypothesized that the Shh pathway might be involved in cognition function in astrocytes.

Naringin (Nrg), aflavonoid, prevents and delays aged-population cognitive dysfunction [8]. Furthermore, Nrg has been reported to attenuate behavioral alterations and cognitive impairment in a mouse model of Alzheimer’s disease [9], as well as in colchicine [10], D-galactose [11] and aluminium [12] induced learning and memory impairment models. It has also been hypothesized that Nrg may improve memory impairment of MHE via the Shh pathway.

In this study, we delineate the role of the Shh signaling pathway in astrocytes in MHE-related memory loss, especially focusing on the effect of elevated DA from cirrhotic liver on Shh pathway in astrocytes. Additonally, we address whether Nrg acts directly on MHE rats to favor memory improvement through the Shh signaling pathway.

## MATERIALS AND METHODS

### MHE models and treatments

A total of 50 Sprague-Dawley rats (experimental animal center of the Chinese Academy of Sciences in Shanghai; weighing 220-250g) were used. Rats were housed under controlled conditions of temperature (24 ± 1°C) and light (12 h light starting at 07:00 am). All experiments were carried out in strict accordance with the recommendations in the Guide for the Care and Use of Laboratory Animals of the State Scientific and Technological Commission. The protocol was approved by the Committee on the Ethics of Animal Experiments of the First Affiliated Hospital of Wenzhou Medical University (Permit Number: 2014-59). All surgery was performed under sodium pentobarbital anesthesia, and all efforts were made to minimize suffering. Before experimenting, all animals have a series of behavioral tests: Y- maze (YM) and water-finding task (WFT). Normal values of these behavioral tests were obtained. Rats were then randomly divided into 2 groups: control group (n=20) and thioacetamid (TAA) group (n=30). MHE was induced by intraperitoneal injection (i.p.) of TAA (200 mg/kg in normal saline; Sigma-aldrich) twice per week for 8 weeks. Behavioral tests were performed for all rats. Criteria for MHE: a) values of YM were lower than 3/4 -fold average normal values; b) Values of WFT were more than 3/2-fold average value normal values; c) Electroencephalograph (EEG) showed no typical slow wave (θ band) of HE [13]. If TAA-treated rats met the criteria of either a) + c) or b) + c), rats were included in the MHE group. After MHE modeling, intracerebroventricular (i.c.v.) injection of Shh protein, human recombinant (merck Millipore; 10 and 50 μg /5 μl in saline) or cyclopamine (Cyc) (Abcam; 1 and 5 μg /5 μl in saline) was performed three times at 48-h intervals, or i.p. injection of Nrg (8 and 80 mg/kg in saline) was performed three times at 7-day intervals. At 24 hrs after the last injection, rats were tested in YM and WFT. After Nrg (80 mg/kg in saline) treatment, Cyc (5 μg /5 μl in saline) was also administered to the rats three times at 48-h intervals by i.c.v. injection. Then rats were anesthetized with intramuscular xylazin (16 mg/kg), the blood was drawn from the aorta abdominalis, and tissues of liver and cerebral cortex were collected.

### DA-treated rat models and treatments

Rats were anesthetized with intramuscular xylazin (16 mg/kg) followed by ketamine (100mg/kg). Injection (i.c.v.) of dopamine hydrochloride (1 μg in 3 μl and 10 μg in 3 μl in saline) was stereotaxically performed in the left lateral ventricles of rats three times at 7-day intervals (anterior -posterior, + 0.3 mm; lateral, 1.0 mm; horizontal, 3.0 mm from the bregma) (n=15). At 24 hrs after the injection, YM and WFT tests were performed. After DA injection, i.c.v. injection of Shh (0.1 μg in 3 μl and 1μg in 3 μl in saline) or Cyc (1 and 5 μg in 5 μl in saline) was performed three times at 48-h intervals, or i.p. injection of Nrg (8 and 80 mg/kg in saline) was performed three times at 7-day intervals. At 24 h after the last injection, YM and WFT were performed. After Nrg (80 mg/kg in saline) treatment, Cyc (5 μg in 5 μl in saline) was also administered to the rats three times at 48-h intervals by i.c.v. injection. Then rats were anesthetized with intramuscular xylazin (16 mg/kg), the blood was drawed from the aortaabdominalis, and cerebral cortex were collected.

### Behavioral tests

The apparatus for YM was made of three arms [14, 15]. The three arms were connected at an angle of 120°. Rats were individually placed at the end of an arm and allowed to explore the maze freely for 8 min. Total arm entries and spontaneous alternation percentage (SA%) were measured. SA% was defined as a ratio of the arm choices that differed from the previous two choices (‘successful choices’) to total choices during the run (‘total entry minus two’ because the first two entries could not be evaluated).

WFT was performed to analyze latent learning or retention of spatial attention of the rats [14, 16]. The testing apparatus consisted of a plastic rectangular open field, and a cubic alcove was attached to the center of one longer wall. A drinking tube was inserted through a hole at the center of the alcove ceiling. A rat was first placed at the near-right corner of the apparatus and allowed to explore it freely for 3 min. Rats were omitted from the analysis when they could not find the tube within the 3 min exploration. In the trial session, rats were again individually placed at the same corner of the apparatus and allowed to find and drink the water in the alcove. The elapsed times were measured until the first entry into the alcove (entry latency, EL), until the first touching/sniffing/licking of the water tube (contacting latency, CL) and until the initiation of drinking from the water tube (drinking latency, DL).

**Figure 1.**
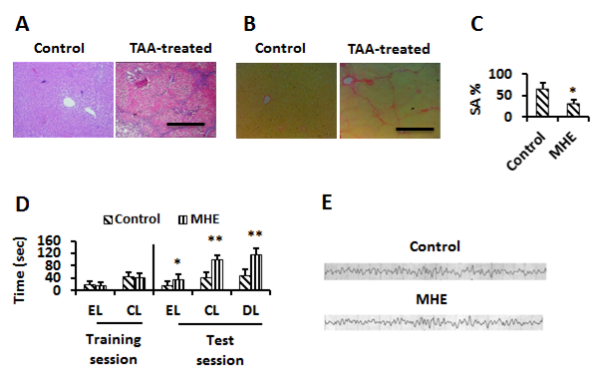
MHE models were successfully established (**A, B**) Liver sections from control and TAA-treated rats were stained by H&E (**A**) and sirius red (**B**). Scale bar, 50 μm. (**C**) Control or MHE rats administrated (i.c.v) with vehicle, 0.1 and1 μg Shh were tested for YM (Spontaneous alternation percentage, SA%). (**D**) Control or MHE rats were tested for WFT (EL, entry latency; CL, contacting latency; DL, drinking latency). (**E**) The cerebral signal of rats observed in the scalp EEG falls in the Alpha (8-13 Hz) range in both of control and MHE rats. Data were shown as mean ± SD. *p <0.05, **p <0.01 vs control group.

### Histopathology

Liver was fixed in 10% formalin for 24 hrs and then parafin-embedded in an automated tissue processor; 5-μm sections were stained with hematoxylin and eosin (H&E) or sirius red and subjected to histopathological examination.

### Determination of DA levels

The analyses were performed with an HPLC system consisting of a model 100A pump (Altex Scientific, Berkeley, CA), an Eicopack MA-ODS reversed-phase column (Eicom Co, Kyoto, Japan), a model 7125 sample injector (Rheodyne, Berkeley, CA), and a model 100 electrochemical detector with a newly developed carbon graphite working electrode WE-3G (Eicom Co.), with the oxidation potential set at 0.7 V vs an Ag/AgCl reference electrode for detecting DA. The HPLC system was assembled so that the analytes separated on the HPLC column flowed first through the ultraviolet absorbance detector, then through the electrochemical detector. Column temperature was maintained at 29? with a temperature-controlled water bath. The mobile phase consisted of an 89/11 (by vol) mixture of methanol with sodium citrate buffer (10 mmol/l, pH 3.1) that contained 10 µmol of EDTA·2Na, 925 µmol of sodium 1-octanesulfonate, and 7.18 mmol of triethylamine per liter. This mobile phase was delivered at a flow rate of 1.0 ml/min. The mobile phase was filtered through a 0.5 µm (pore size) filter membrane (Toyo Roshi Co., Ltd., Tokyo, Japan) under reduced pressure. All chromatograms were recorded and the areas under the peaks of the respective analytes were integrated with a model D-2000 Chromato-Integrator (Hitachi Ltd., Tokyo, Japan).

### Cell culture and treatments

Primary cortical astrocytes (PCAs) were prepared from 1-day-old Sprague- Dawley rat pups [17]. Tissues of cerebral cortex were dissociated into a cell suspension using mechanical digestion. Cells were plated in 75 cm^2^ tissue culture flasks at a concentration of 15 ×10^6^ cells in 11 ml of 1% serum-containing DMEM/F12 medium, incubating for 72 hrs. The medium was changed every 72 hrs. After incubating the primary cultures for 7 days, the medium was changed completely (11 ml). Flasks were placed on a shaker platform in a horizontal position, and were shaken at 200 *g* for 18 hrs at 37°C to separate the oligodendrocytes from the astrocytes. Cells were then poured into a new 75 cm^2^ flask, incubated for 7 days and plated in six-well plates. Astrocytes were exposed to dopamine hydrochloride (final concentration of 1 and 10 μM in 1% DMSO) 24 hrs with and without 6 hr pretreatment with Shh (0.3 and 3 μM in 1% DMSO), Cyc (1 and 10 μM in 1% DMSO), Nrg (5 and 50 μM in 1% DMSO), or Nrg (50 μM in 1% DMSO) +Cyc (10 μM in 1% DMSO).

**Figure 2.**
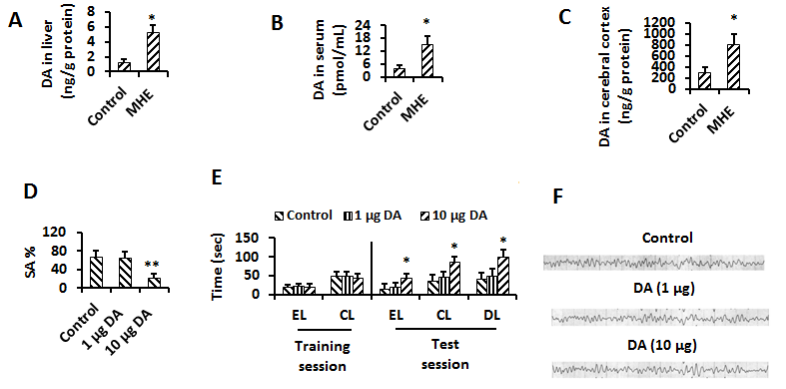
The intracranial elevated DA induced the memory impairment (**A-C**) Liver/cerebral cortex homogenates and serum extract from control or MHE group were analysed for DA concentration by HPLC. (**D**) Control, 1μg or 10 μg DA-injected (i.c.v) rats were tested for YM (Spontaneous alternation percentage, SA%). (**E**) Control, 1μg or 10 μg DA- injected (i.c.v) rats were tested for WFT (EL, entry latency; CL, contacting latency; DL, drinking latency). (**F**) No Theta (4-7 Hz) or Delta (< 4 Hz) bands (slow wave) were seen in EEG of DA (i.c.v.) -treated rats. Data were shown as mean ± SD. *p <0.05, **p <0.01 vs controls; ^#^*p* <0.05, ^# #^*p* <0.01 vs MHE groups.

### Reverse transcription-PCR(RT-PCR) and Real-time Quantitative PCR (qPCR)

Total RNA was isolated using the Qiagen RNA-Easy kit according to the manufacturer’s protocol. cDNA was created using oligo (dT), dNTP, 0.1 M DTT, Moloney murine leukemia virus reverse transcriptase, RNaseOUT, and 5× FS Buffer (all from Invitrogen) and amplified with PCR Master Mix (Promega). The following primers (Invitrogen) for murine genes: BDNF-5′-GCGGACC CATGGGACTCT-3′ (Forward) and 5′-CTGCTGCTGT AGTGACCGA-3′ (Reverse); NT-3-5′-GAGAGGCC ACCAGGTCAGAGTTCCA-3′ (Forward) and 5′-GTCA TCAATCCCCCTGCAACCGTTT-3′ (Reverse); GAP DH-5′-ACCCAGAAGACTGTGGATGG-3′(Forward) and 5′-ACACATTGGGGGTAGGAACA-3′(Reverse).

Amplified products were electrophoresed on 2% agarose gels, visualized by EtBr staining, and normalized to β-actin. qPCR was performed using the ABI-Prism7700 sequence detection system (Applied Biosystems), iTaq™ Fast Supermix with ROX (Bio-Rad) and 6-carboxyfluorescein-labeled TAAR1, EAAT2, GluR1, BDNF, CaNA, NFAT3, NT-3, and β-actin primers (Integrated DNA Technologies). The mRNA expression was analyzed using the relative 2(-∆∆C(T)) method.

**Figure 3.**
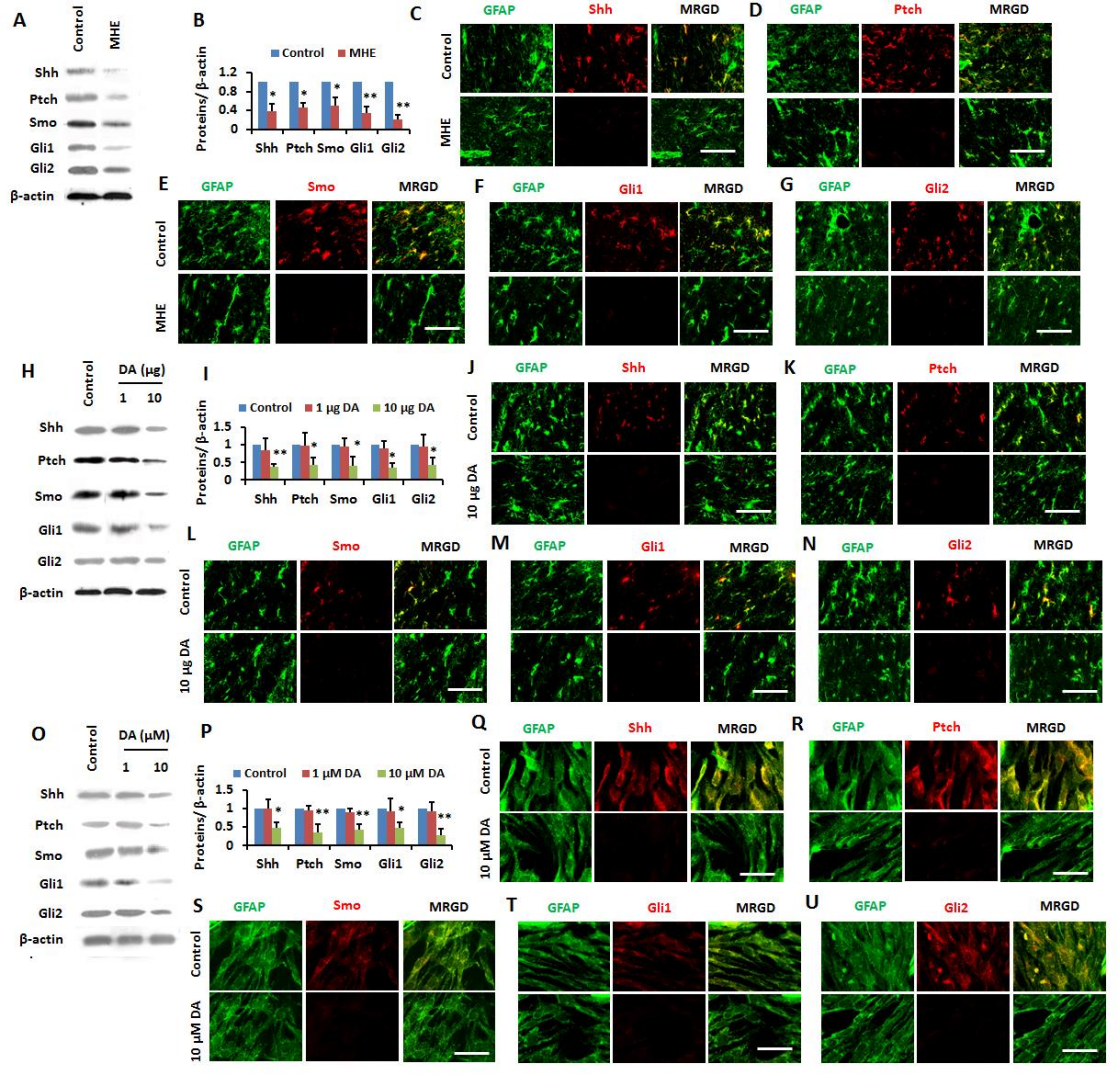
The Shh pathway was inactivated in MHE by DA burden (**A** and **B**) Cortical homogenates from control and MHE rats were subjected to immunoblotting with antibodies against Shh, Ptch, Smo, Gli1, Gli2 and β-actin and subsequent densitometry (**B**). (**C-G**) Free floating coronal sections from cortexes of control or MHE rats were immunostained for Shh (**C**)/ Ptch (**D**)/ Smo (**E**)/ Gli1 (**F**)/ Gli2 (**G**) (red), GFAP (green). Scale bar, 25 μm. MRGD, merged image. (**H** and **I**) Cortical homogenates from control, 1 μg or 10 μg DA- injected (i.c.v) rats were subjected to immunoblotting with antibodies against Shh, Ptch, Smo, Gli1, Gli2 and β-actin, and subsequent densitometry (**I**). (**J-N**) Free floating coronal sections from cortexes of control or 10 μg DA-injected (i.c.v) rats were immunostained for Shh (**J**)/ Ptch (**K**)/ Smo (I)/ Gli1(**M**)/ Gli2 (**N**) (red), GFAP (green). Scale bar, 25 μm. MRGD, merged image. (**O** and **P**) Cells were treated with different doses of DA for 24 hrs before being subjected to immunoblotting with antibodies against Shh, Ptch, Smo, Gli1, Gli2 and β-actin, and subsequent densitometry (**P**). (**Q-U**) Cells were treated with different doses of DA for 24 hrs and immunostained for Shh (**Q**)/ Ptch (**R**)/ Smo (**S**)/ Gli1(**T**)/ Gli2 (**U**) (red), GFAP (green). Scale bar, 50 μm. MRGD, merged image. Data were shown as mean ± SD. *p <0.05, **p <0.01 vs controls.

**Figure 4.**
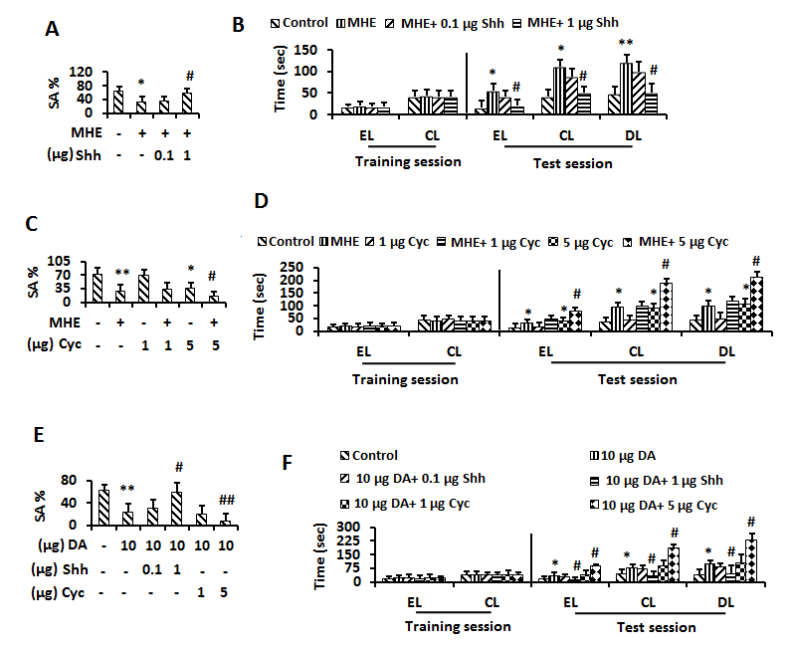
The inactivation of Shh pathway induced memory impairment in MHE (**A**) Control or MHE rats administrated (i.c.v) with vehicle, 0.1 and1 μg Shh were tested for YM. Data were shown as mean ± SD. *p <0.05, **p <0.01 vs control group; ^#^*p*<0.05, ^# #^*p*<0.01 vs MHE group. (**B**) Control or MHE rats administrated (i.c.v) with vehicle, 0.1 and 1 μg Shh were tested for WFT (EL, entry latency; CL, contacting latency; DL, drinking latency). Data were shown as mean ± SD. *p <0.05, **p <0.01 vs control group; ^#^*p*<0.05, ^##^*p*<0.01 vs MHE group. (**C**) Control or MHE rats administrated (i.c.v) with vehicle, 1 and 5 μg Cyc were tested for YM. Data were shown as mean± SD. *p <0.05, **p <0.01 vs control group; ^#^*p*<0.05, ^# #^*p*<0.01 vs MHE group. (**D**) Control or MHE rats administrated (i.c.v) with vehicle, 1 and 5 μg Cyc were tested for WFT. Data were shown as mean ± SD. *p <0.05, **p <0.01 vs control group; ^#^*p*<0.05, ^# #^*p*<0.01 vs MHE group. (**E**) Control, 1μg or 10 μg DA- injected (i.c.v) rats administrated with vehicle, 0.1 and 1 μg Shh, 1 and 5 μg Cyc were tested for YM. Data were shown as mean ± SD. *p <0.05, **p <0.01 vs control group; ^#^*p*<0.05, ^# #^*p*<0.01 vs DA (10 μg) - treated group. (**F**) Control, 1 μg or 10 μg DA- injected (i.c.v) rats administrated with vehicle, 0.1 and 1 μg Shh, 1 and 5 μg Cyc were tested for WFT. Data were shown as mean ± SD. *p <0.05, **p <0.01 vs control group; ^#^*p*<0.05, ^# #^*p*<0.01 vs DA (10 μg) - treated rats group.

### Immunoblotting

Cerebral cortex tissues or PCAs were harvested in a lysis buffer (50 mM Tris HCl (pH7.4), 150 mM NaCl, 1% Triton-X100, 1 mM PMSF, 2μg/ml aprotinin, 2ug/ml leupeptin, 1.5 mM EDTA) (Sigma-Aldrich). The total amount of protein was determined by bicinchoninic acid (BCA) protein assay (Amresco). Proteins (50 μg) were separated by 10% SDS-PAGE and electroblotted to PVDF membrane, which were blocked by incubation in 5% non-fat dry milk dissolved in TBS-T (150 mM NaCl, 50 mM Tris, 0.05% Tween-20). Following transfer, proteins were probed using a primary antibody: Shh (1:2000), Ptch (1:1000), Smo (1:1000), Gli1 (1:400), Gli2 (1:1000), BDNF (1:1000), NT3 (1:500), or β-actin (1:5000), Abcam. Then horseradish peroxidase-conjugated secondary antibody was used. After extensive washing, protein bands detected by antibodies were visualized by ECL reagent (Pierce) after exposure on Kodak BioMax film (Kodak).

**Figure 5.**
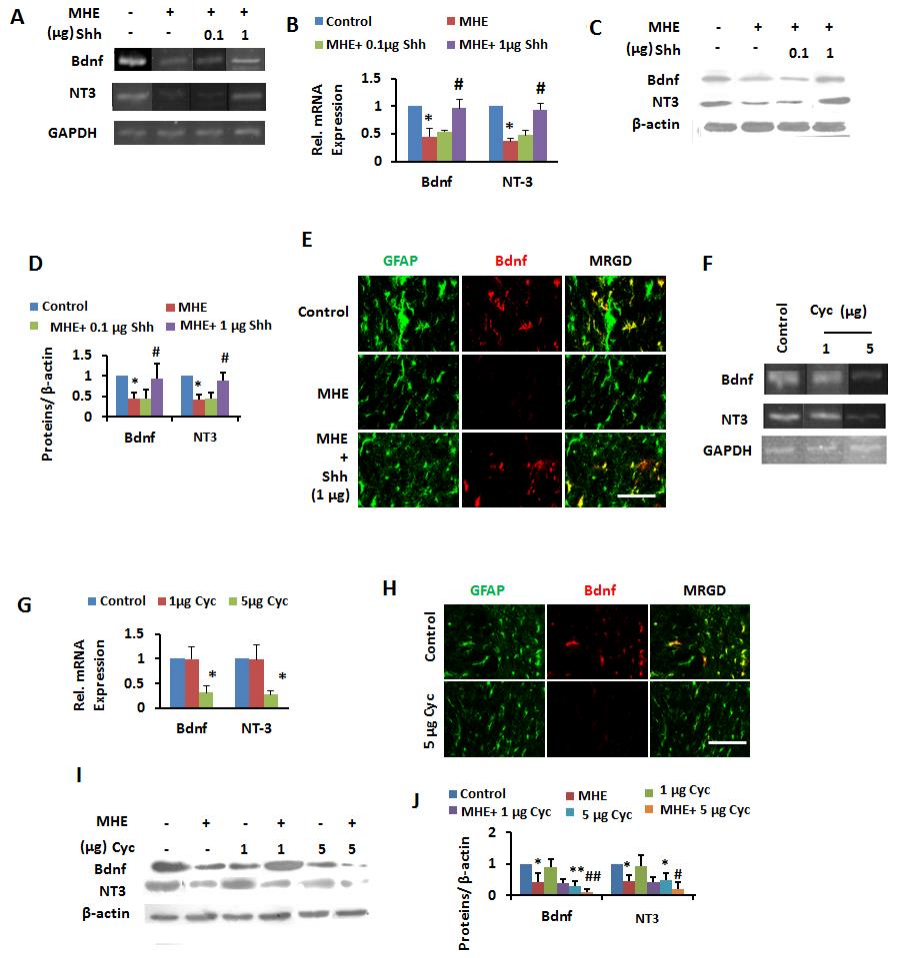
The inactivation of Shh pathway induced downregulation of memory -related neurotrophins in MHE (**A** and **B**) Cortical lysate from control and MHE rats administrated with vehicle, 0.1 and 1 μg Shh were analyzed for BDNF, NT3, GAPDH mRNAs by RT-PCR (**A**) and qPCR (**B**). (**C** and **D**) Cortical homogenates from control and MHE rats administrated (i.c.v) with vehicle, 0.1 and 1 μg Shh were subjected to immunoblotting with antibodies against BDNF, NT3, and β-actin and subsequent densitometry (**D**). Data were shown as mean ± SD. *p <0.05, **p <0.01 vs control group; ^#^*p*<0.05, ^##^*p*<0.01 vs MHE group. (**E**) Free floating coronal sections from cerebral cortexes of MHE rats administrated (i.c.v) with vehicle, 1 μg Shh were immunostained for BDNF (red), GFAP (green). Scale bar, 25 μm. MRGD, merged image. (**F-G**) Cortical lysate from control rats administrated with vehicle, 1 and 5 μg Cyc were analyzed for BDNF, NT3, GAPDH mRNAs by RT-PCR (**F**) and qPCR (**G**). (**H**) Free floating coronal sections from cortexes of control rats administrated (i.c.v) with vehicle, 1 and 5 μg Cyc were immunostained for BDNF (red), GFAP (green). Scale bar, 25 μm. MRGD, merged image. (**I** and **J**) Cortical homogenates from MHE rats administrated (i.c.v) with vehicle, 5 μg Cyc were subjected to immunoblotting with antibodies against BDNF, NT3, and β-actin and subsequent densitometry (**J**). Data were shown as mean ± SD. *p <0.05, **p <0.01 vs control group; ^#^*p*<0.05, ^##^*p*<0.01 vs MHE group.

**Figure 6.**
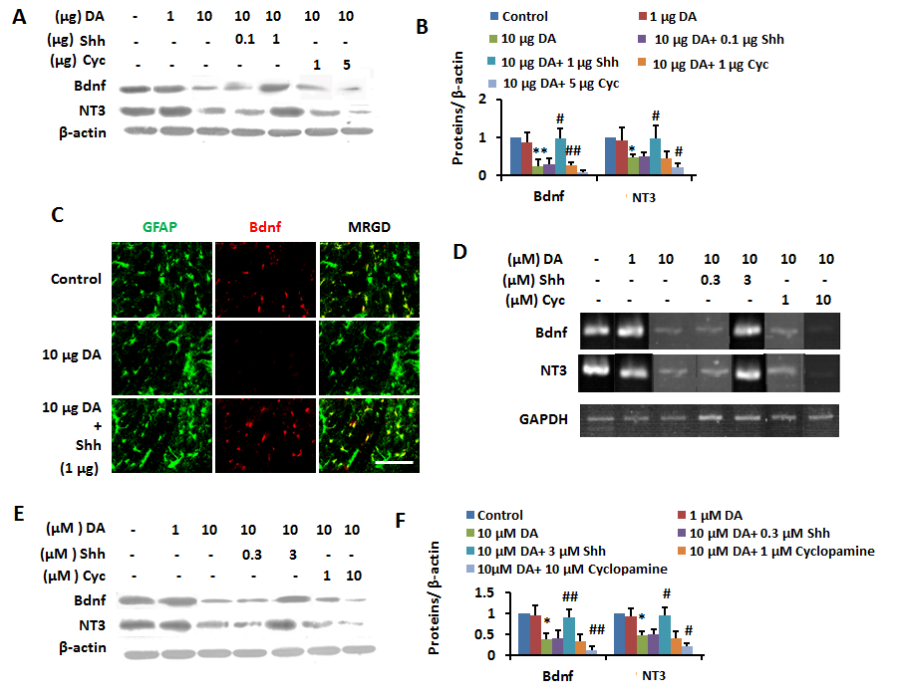
DA-induced inactivation of Shh pathway downregulated neurotrophins (**A** and **B**) Cortical homogenates from control, 1 μg or 10 μg DA-injected (i.c.v) rats administrated with vehicle, 0.1 and 1 μg Shh, 1 and 5 μg Cyc were subjected to immunoblotting with antibodies against BDNF, NT3, and β-actin and subsequent densitometry (**B**). Data were shown as mean ± SD. *p <0.05, **p <0.01 vs control group; ^#^*p*<0.05, ^##^*p*<0.01 vs DA (10μg) - treated group. (**C**) Free floating coronal sections from cortexes of 10μg DA-injected (i.c.v) rats administrated (i.c.v) with vehicle, 1 μg Shh, 5 μg Cyc were immunostained for Bdnf (red), GFAP (green). scale bar, 25 μm. MRGD, merged image. (**D**) Cells were treated with different doses of DA alone or after preincubation with 0.3 and 3 μM Shh, 1 and 10 μM Cyc for 24h and analyzed for Bdnf, NT3, GAPDH mRNAs by RT-PCR. (**E** and **F**) Cells were treated with different doses of DA alone or after preincubation with 0.3 and 3 μM Shh, 1 and 10 μM Cyc for 24h before being subjected to immunoblotting with antibodies against Bdnf, NT3, and β-actin and subsequent densitometry (**F**). Data were shown as mean± SD. *p <0.05, **p <0.01 vs control group; ^#^*p*<0.05, ^##^*p*<0.01 vs DA (10μM) - treated group.

### Double-labeled fluorescent staining

For cerebral cortex tissues: four-micron frozen cerebral cortex sections fixed in acetone or 4% formaldehyde were blocked for endogenous peroxidase activity with 0.03% H_2_O_2_ if appropriate. For PCAs: PCAs were seeded and cultured on glass coverslips precoated with 0.01% poly-L-lysine (Sigma-Aldrich) for 1 hr. After the cells were treated with DA (final concentration of 1, 5 and 10 μM) for 24 hrs, they were fixed with 4 % paraformaldehyde for 30 min and then treated with 0.1% Triton X-100 for 10 min at room temperature.

Blocking was achieved with PBS containing 5% normal goat serum for 1 h at room temperature. Sections were then incubated overnight at 4°C with the following primary antibodies: Shh (1:500), Ptch (1:1000), Smo (1:200), Gli1 (1:200), Gli2 (1:200), BDNF (1:200), GFAP (1:1000), Abcam. Binding of primary antibodies was detected by incubating the sections for 30 min with Alexa Fluor 488 (green)/Alexa Fluor 594 (red) conjugated secondary antibody.

### Statistical analysis

Data were presented as mean ± SEM. The statistical significance between group comparisons was determined by one-way analysis of variance (ANOVA), followed by post hoc Tukey’s multiple comparison test. Values of *p* < 0.05 or *p* < 0.01 were statistically significant.

## RESULTS

### Establishment of MHE models

As shown in Fig. 1A and B, regenerating hepatic nodules and inflammatory cell infiltration composed of lymphocytes and plasma cells were present based on HE staining (Fig. 1A) and fibrous septa formation was observed following Sirius red staining (Fig. 1B) in the liver of TAA-treated rats. These observations suggested that the liver cirrhosis model was successfully established by TAAi.p. injection.

Rats were then subjected to behavioral tests and an EEG test. Of the TAA-treated rats, 24/30 exhibited loss of spatial working memory based on a significant decrease in spontaneous alternation percentage (SA%) in a YM test (Fig. 1C). In a WFT, 22/30 TAA-treated rats exhibited latent memory deficits based on a significant delay in EL, DL and CL (Fig. 1D). Accordingly, there were 21 TAA-treated rats with cognitive impairment; 20 displayed an alpha (8-13 Hz) band (normal band) and no θ (4-7 Hz) band (typical slow wave of HE) in the EEG tests (Fig. 1E), suggesting that 20/21 showed MHE.

### Elevated intracranial DA causes memory impairment

Elevation of intracranial DA levels was found in MHE rats in our previous study [3], suggesting the blood-brain barrier permeance of elevated DA from cirrhotic liver in MHE rats. We confirmed increased levels of DA in the liver (Fig. 2A), serum (Fig. 2B) and cerebral cortex (Fig. 2C) in MHE rats compared with control rats.

Then we i.c.v. injected DA (1 and 10 μg) into normal rats. The rats were subjected to behavioral tests and an EEG test as well. In YM, a significant decreased of SA% was observed in rats treated with 10 µg DA (Fig. 2D). WFT identified a significant delay in EL, CL and DL in the rats treated with 10 µg DA (Fig. 2E). An alpha band and no θ band in the EEG tests were detectable in DA-treated rats (Fig. 2F). These observations confirmed that the MHE can be induced by excess DA.

**Figure 7.**
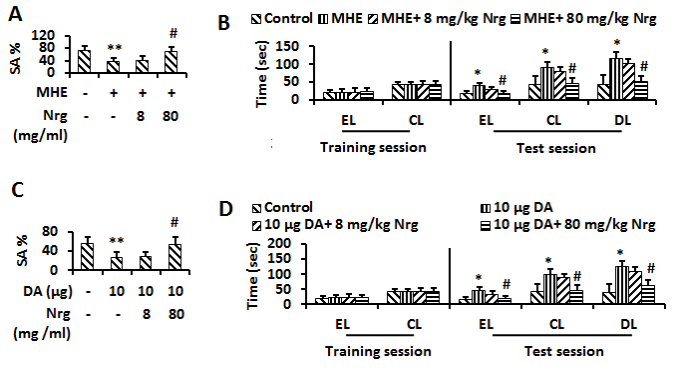
Nrg improved memory impairment (**A**) Control or MHE rats administrated (i.p.) with vehicle, 8 and 80 mg/kg Nrg were tested for YM. Data were shown as mean ± SD. *p <0.05, **p <0.01 vs control group; ^#^*p*<0.05, ^##^*p*<0.01 vs MHE group. (**B**) Control or MHE rats administrated (i.p.) with vehicle, 8 and 80 mg/kg Nrg were tested for WFT (entry latency (EL)/ contacting latency (CL)/ drinking latency (DL)). Data were shown as mean ± SD. *p <0.05, **p <0.01 vs control group; ^#^*p*<0.05, ^##^*p*<0.01 vs MHE group. (C) Control, 10 μg DA- injected (i.c.v) rats administrated with vehicle, 8 and 80 mg/kg Nrg were tested for YM. Data were shown as mean ± SD. *p <0.05, **p <0.01 vs control group; ^#^*p*<0.05, ^##^*p*<0.01 vs DA (10μg) -treated group. (d) Control, 10μg DA- injected (i.c.v) rats administrated with vehicle, 8 and 80 mg/kg Nrg were tested for WFT. Data were shown as mean± SD. *p <0.05, **p <0.01 vs control group; ^#^*p*<0.05, ^##^*p*<0.01 vs DA (10μg) - treated group.

**Figure 8.**
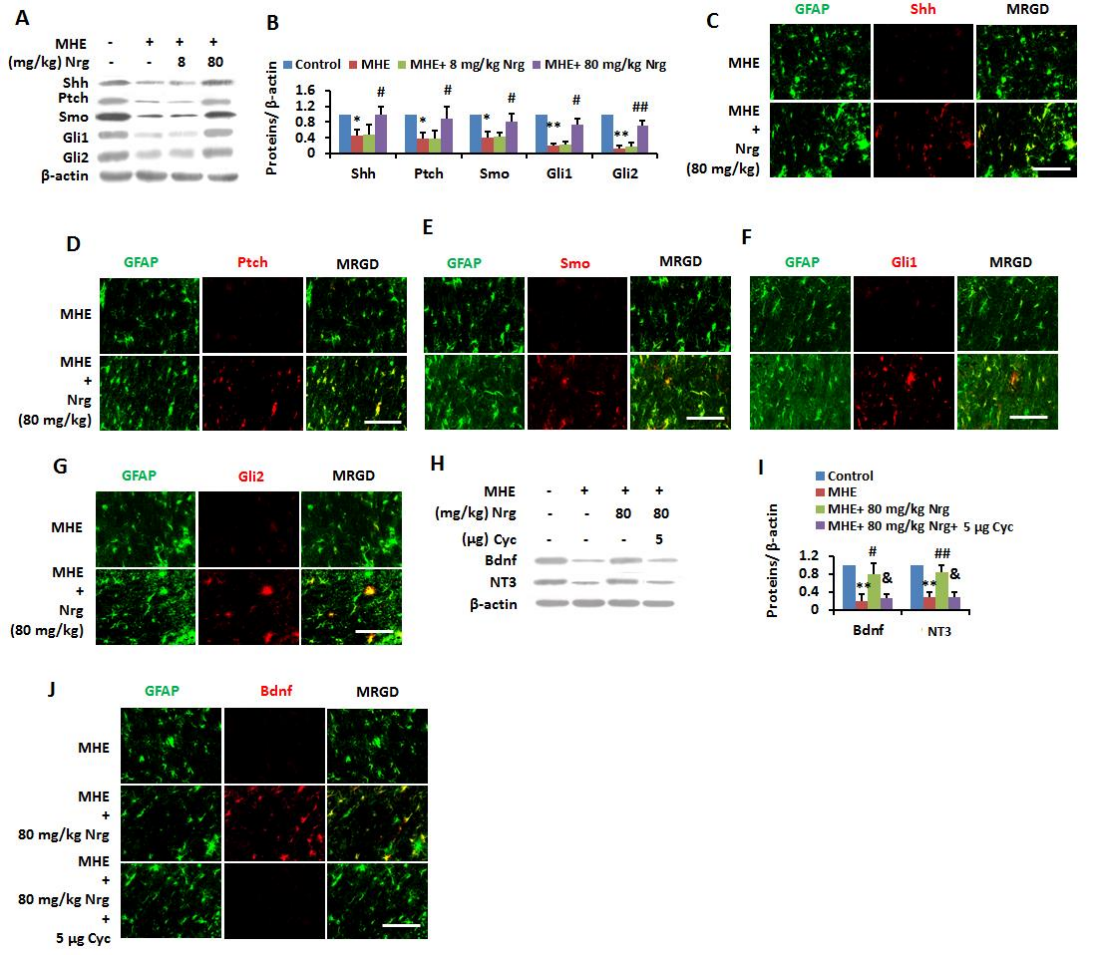
Nrg improved the activation of Shh pathway in MHE (**A** and **B**) Cortical homogenates from control and MHE rats administrated (i.p.) with vehicle, 8 and 80 mg/kg Nrg were subjected to immunoblotting with antibodies against Shh, Ptch, Smo, Gli1, Gli2 and β-actin and subsequent densitometry (**B**). Data were shown as mean ± SD. *p <0.05, **p <0.01 vs control group; ^#^*p*<0.05, ^##^*p*<0.01 vs MHE group. (**C-G**) Free floating coronal sections from cortexes of MHE rats administrated (i.p.) with vehicle, 80 mg/kg Nrg were immunostained for Shh (**C**)/Ptch (**D**)/Smo (**E**)/Gli1 (**F**)/Gli2 (**G**) (red), GFAP (green). Scale bar, 25 μm. MRGD, merged image. (**H, I**) Cortical homogenates from control and MHE rats administrated (i.c.v) with vehicle, 80 mg/kg Nrg and 80 mg/kg Nrg + 5 μg Cyc were subjected to immunoblotting with antibodies against BDNF, NT3, and β-actin and subsequent densitometry (**I**). Data wereshown as mean ± SD. *p <0.05, **p <0.01 vs control group; ^#^*p*<0.05, ^##^*p*<0.01 vs MHE group; ^&^*p*<0.05, ^&&^*p*<0.01 vs MHE+ Nrg group. (**J**) Free floating coronal sections from cerebral cortexes of MHE rats administrated with vehicle, 80 mg/kg Nrg and 80 mg/kg Nrg + 5 μg/ml Cyc were immunostained for Bdnf (red), GFAP (green). Scale bar, 25 μm. MRGD, merged image.

### Inactivation of Shh signaling pathwayinduced by DA burden in MHE

Given that Shh has neuroprotective effects [18] and maintains astrocyte function in the adult brain [19], we sought to determine whether activation of the Shh signaling pathway was altered in astrocytes in the cerebral cortex of MHE rats. To investigate if changes in expression of proteins were involved in Shh signaling pathway in cerebral cortex of MHE rats, we performed double immunofluorescence (IF) and immunoblotting (IB) testes. The results demonstrated that the expression of Shh, Ptch, Smo, Gli1, and Gli2 was strongly decreased in the cerebral cortex based on IB (Fig. 3A and B). As indicated by Fig. 3C-G, MHE rats displayed a reduced expression of Shh, Ptch, Smo, Gli1, and Gli2 in cerebral cortex compared to control rats based on double IF staining.

To confirm whether the elevated intracranial DA in MHE led to inactivation of the Shh pathway, we used rats injected with DA (1 and 10 μg, i.c.v.) to examine the expression of Shh, Ptch, Smo, Gli1, and Gli2 in cerebral cortex. Based on IB analysis, we found a decreased expression of these proteins in the cerebral cortex of rats treated with 10 μg DA (Fig. 3H and I). Double IF staining with antibodies against Shh, Ptch, Smo, Gli1 or Gli2 and an anti-GFAP antibody (anastrocytic marker) confirmed the downregulation of these proteins in astrocytes of cerebral cortex of DA-treated rats compared to control rats (Fig. 3J and N). These results implicated the intracranial DA burden in the inactivation of the Shh signaling in MHE.

I addition, we further examined the effect of DA on the Shh signaling pathway in PCA *in vitro*. Downregulation of Shh, Ptch, Smo, Gli1 and Gli2 was induced only by 10 μM DA treatment, but not by lower dose of DA (1 μM) based on IB (Fig. 3O and P) and IF staining (Fig. 3Q-U). These results suggested that the levels of these proteins in the astrocytes were reciprocally correlated with the brain DA burden.

**Figure 9.**
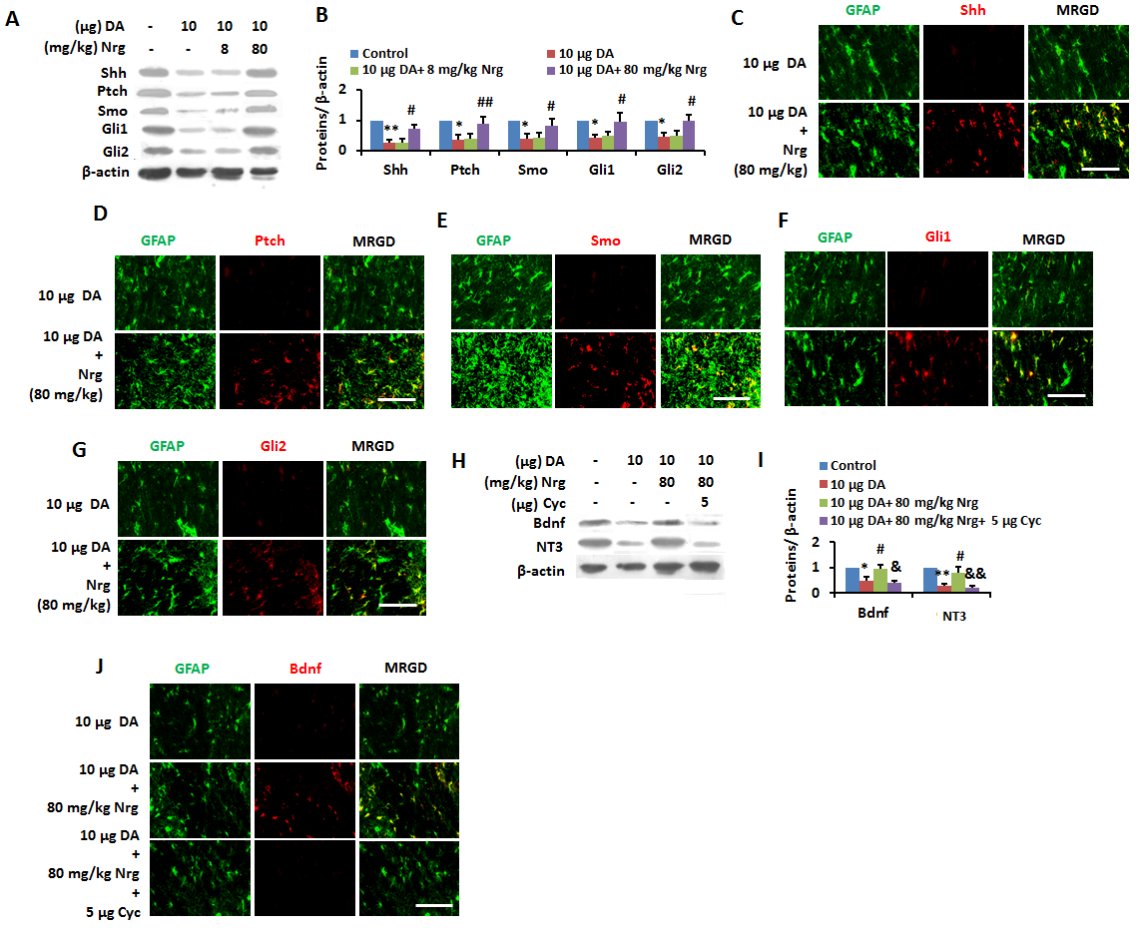
Nrg reversed DA-induced inactivation of Shh pathway *in vivo* (**A** and **B**) Control, 10 μg DA- injected (i.c.v) rats administrated with vehicle, 8 and 80 mg/kg Nrg were subjected to immunoblotting with antibodies against Shh, Ptch, Smo, Gli1, Gli2 and β-actin and subsequent densitometry (**B**). Data wereshown as mean ± SD. *p <0.05, **p <0.01 vs control group; ^#^*p*<0.05, ^##^*p*<0.01 vs DA (10 μg) - treated group. (**C-G**) Free floating coronal sections from cerebral cortexes of 10 μg DA- injected (i.c.v) rats administrated with vehicle, 80 mg/kg Nrg were immunostained for Shh (**C**)/ Ptch (D)/ Smo (**E**)/ Gli1 (**F**)/ Gli2 (**G**) (red), GFAP (green). Scale bar, 25 μm. MRGD, merged image. (**H, I**) Cortical homogenates from Control, 10 μg DA-injected (i.c.v) rats administrated with vehicle, 80 mg/kg Nrg and 80 mg/kg Nrg + 5 μg Cyc were subjected to immunoblotting with antibodies against BDNF, NT3, and β-actin and subsequent densitometry (**I**). Data wereshown as mean ± SD. *p <0.05, **p <0.01 vs control group; ^#^*p*<0.05, ^##^*p*<0.01 vs DA (10μg) - treated group; ^&^*p*<0.05, ^&&^*p*<0.01 vs 10 μg DA+ 80 mg/kg Nrg-treated group. (**J**) Free floating coronal sections from cortexes of 10 μg DA- injected (i.c.v) rats administrated with vehicle, 80 mg/kg Nrg and 80 mg/kg Nrg + 5 μgCyc were immunostained for BDNF (red), GFAP (green). Scale bar, 25 μm. MRGD, merged image.

### Involvement of the Shh pathway in memory impairment in MHE rats

We next attempted to elucidate memory-related downstream targets of the Shh pathway by using the Shh signaling activator Shh and the inhibitor Cyc. We i.c.v. injected Shh (0.1 and 1μg) into MHE rats. Rats were tested in YM and WFT. SA% of MHE rats, which was lower than that of wild type littermates before treatment, recovered to normal levels after treatment with 1 μg Shh (Fig. 4A). The significant delays in EL, CL and DL were reversed to normal levels detected in wild type with 1 μg Shh treatment (Fig. 4B). We i.c.v. injected Cyc (1 and 5 μg) into normal and MHE rats. The rats were then tested in YM and WFT. SA% in YM in the 5μg Cyc-injected rats was significantly lower than that of controls, and 5μg Cyc injected MHE rats showed amplified loss of spatial working memory compared to MHE rats (Fig. 4C). In WFT, a significant delay in EL, CL and DL in rats injected with 5 µg Cyc was detected compared with controls, and similarly treated MHE rats also showed amplified latent memory deficits compared to MHE rats (Fig. 4D).

We then investigated whether DA blocked memory function by inactivation of the Shh pathway. We i.c.v. injected Shh (0.1 and 1 μg) and Cyc (1 and 5 μg) into DA-treated rats and tested them in YM and WFT. In YM, the SA% was decreased in the rats treated with 10 µg DA, which was reversed by injection of 1 μg Shh and amplified by injection of 5 μg Cyc (Fig. 4E). In WFT, the delay in EL, CL and DL in the rats was reversed by Shh, but amplified by Cyc (Fig. 4F).

### Inactivation of Shh pathway signaling to neurotrophinsin MHE

RT-PCR (Fig. 5A) and qPCR (Fig. 5B) revealed significant decreases in BDNF and NT3 mRNA levels in cerebral cortex of MHE rats compared with controls. Based on IB analysis (Fig. 5C and D), administration of 1 μg Shh significantly counteracted the downregulation of these proteins in cerebral cortex of MHE rats. We also found recovery of BDNF and NT3 levels in astrocytes following administration of 1 µg Shh in cerebral cortex of MHE rats based on IF staining (Fig. 5E).

Based on RT-PCR (Fig. 5F) and qPCR (Fig. 5G), i.c.v. injection of 5 μg Cyc into normal rats induced the decreases in BDNF and NT3 mRNA levels in cerebral cortex. Double IF staining also revealed weak expression of these proteins in astrocytes in cerebral cortex following Cyc administration (Fig. 5H). Furthermore, based on IB staining, though these proteins were largely absent in cerebral cortex of MHE rats, their levels were amplified by administration of 5 μg Cyc (Fig. 5 I and J). These observations indicated that the Shh pathway signals to neurotrophins and that the inactivation of the Shh-neurotrophins pathway is responsible for the memory impairment in MHE rats.

Furthermore, IB analysis (Fig. 6A-B) revealed that BDNF and NT3 levels were reduced in cerebral cortex of DA-treated rats and this effect was diminished by Shh and enhanced by Cyc. As also determined by IF staining, Shh administration induced the weak expression of these proteins in astrocytes in cerebral cortex of DA-treated rats to normal levels (Fig. 6C). We then added Shh (0.3 and 3 μM) and Cyc (1 and 10 μM) to PCA treated with 10 µM DA. Semi-quantitative RT-PCR (Fig. 6D) revealed that DA-induced decreases in BDNF and NT3 mRNA levels in PCA were abrogated by 3 μM Shh treatment; while they were enhanced by addition of 10 μM Cyc. Similarly, based on IB, the reduction in BDNF and NT3 levels in response to 10 μM DA was abolished by 3 μM Shh but strongly augmented by 10 μM Cyc (Fig. 6E and F). These results indicated that DA-induced memory impairment could be attributed to inhibition of the Shh-IEG pathway.

### Nrg improves memory impairment

Given that Nrg was found to improve cognitive deficits, and Shh pathway regulated memory ability in MHE rats, we examined whether Nrg could improve memory impairment of MHE rats by activation of Shh pathway. We first performed a behavioral test for MHE rats injected with 8 and 80 mg/kg Nrg. In YM, 80 mg/kg Nrg caused recovery resulting from the decrease of SA% in the MHE rats to normal levels (Fig. 7A). In WFT, the delay in EL, CL and DL in MHE rats also recovered to normal levels following 80 mg/kg Nrg administration (Fig. 7B). Additionally, we examined whether Nrg could improve DA-induced inactivation of Shh pathway. We first performed behavioral tests for DA-treated rats i.p. injected with 8 and 80 mg/kg Nrg. In YM, loss of spatial working memory in 0 μg DA-treated rats was reversed by 80 mg/kg Nrg administration (Fig. 7C). In WFT, the significant delay in EL, CL and DL in DA-treated rats recovered to normal levels following administration of 80 mg/kg Nrg (Fig. 7D).

### Nrg activates Shh pathway in MHE rats

We examined whether Nrg could stimulate the Shh pathway signaling to neurotrophins. IB staining revealed the recovery of downregulated Shh, Ptch, Smo, Gli1 and Gli2 in cerebral cortex of MHE ratsto normal levels by administration of 80 mg/kg Nrg (Fig. 8 A and B). In parallel, based on double IF, we observed that the lower expression of these proteins in astrocytes in cerebral cortex of MHE rats was reversed by administration of 80 mg/kg Nrg (Fig. 8C-G). We also found that the downregulation of BDNF and NT3 in cerebral cortex of MHE rats was reversed to normal levels by 80 mg/kg Nrg, whereas the effect of Nrg was abolished by 5 μg Cyc based on IB analysis (Fig. 8 H and I) and IF staining (Fig. 8J).

**Figure 10.**
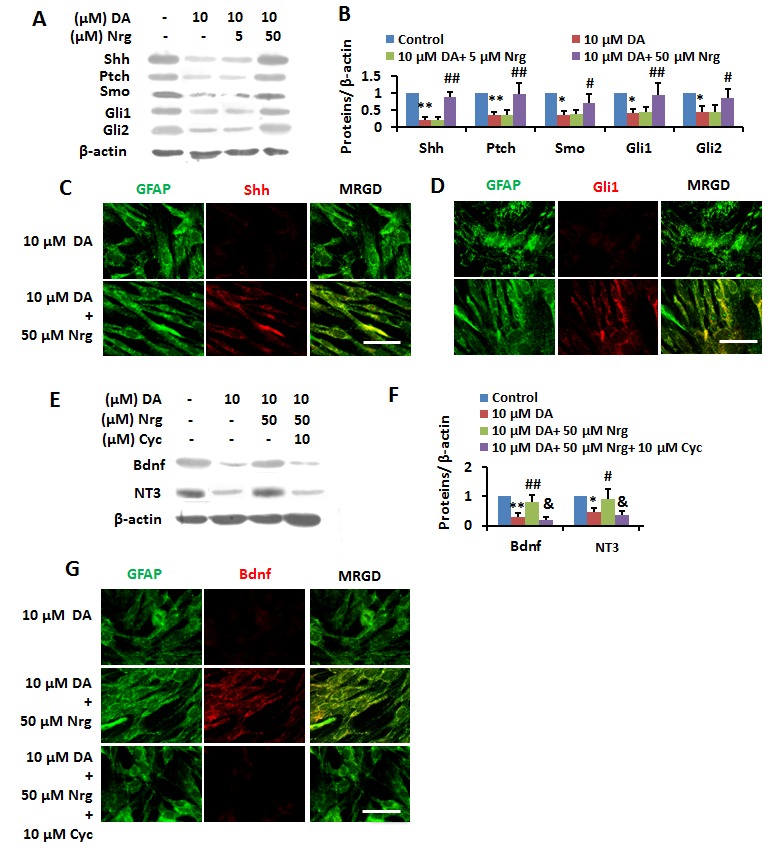
Nrg reversed DA-induced inactivation of Shh pathway in primary cultured astrocytes (PCAs). (**A** and **B**) Cells were treated with different doses of DA alone or after preincubation with vehicle, 5 and 50 μM Nrg for 24 hrs were subjected to immunoblotting with antibodies against Shh, Ptch, Smo, Gli1, Gli2 and β-actin and subsequent densitometry (**B**). Data wereshown as mean ± SD. *p <0.05, **p <0.01 vs control group; ^#^*p*<0.05, ^##^*p*<0.01 vs DA (10 μM) -treated group. (**C** and **D**) Cells were treated with different doses of DA alone or after preincubation with vehicle, 5 and 50 μM Nrg for 24 hrs were immunostained for Shh (**C**)/Gli1 (**D**) (red), GFAP (green). Scale bar, 25 μm. MRGD, merged image. (**E** and **F**) Cells were treated with different doses of DA alone or after preincubation with vehicle, 50 μM Nrg and 50 μM Nrg +10 μM Cyc for 24 hrs were subjected to immunoblotting with antibodies against BDNF, NT3, and β-actin and subsequent densitometry (**F**). Data wereshown as mean ± SD. *p <0.05, **p <0.01 vs control group; ^#^*p*<0.05, ^##^*p*<0.01 vs DA (10 μM)-treated group; ^&^*p*<0.05, ^&&^*p*<0.01 vs 10 μM DA + 50 μM Nrg-treated group. (**G**) Cells were treated with different doses of DA alone or after preincubation with vehicle, 50 μM Nrg and 50 μM Nrg +10 μM Cyc for 24 hrs were immunostained for BDNF (red), GFAP (green). Scale bar, 25 μm. MRGD, merged image.

The above results indicated that Nrg improved memory impairment of MHE by activation of the Shh-neurotrophins signaling pathway. We then investigated the activation of Nrg on Shh pathway signaling to neurotrophinsin cerebral cortex of DA-treated rats. We found that administration of 80 mg/kg Nrg improved the expression of Shh, Ptch, Smo, Gli1 and Gli2 in cerebral cortex of DA-treated rats (Fig. 9A and B), and based on IF staining, it restored the expression of these proteins in astrocytes (Fig. 9C-G). Furthermore, IB analysis (Fig. 9H and I) and IF staining (Fig. 9J) demonstrated that decreased levels of BDNF and NT3 in cerebral cortex of DA-treated rats were recovered to the normal levels after administration of 80 mg/kg Nrg, while the effect of Nrg could be diminished by 5μg Cyc.

Finally, we investigated the effect of Nrgon Shh pathway signaling to neurotrophins *in vitro*. As expected, the 10 μM DA-induced downregulation of Shh, Ptch, Smo, Gli1 and Gli2 in PCA was substantially reversed by addition of 50 μM Nrg based on IB analysis (Fig. 10A and B) and IF staining (Fig. 10C and D). Moreover, as determined by IB analysis (Fig. 10E and F) and IF staining (Fig. 10G), the DA-mediated decreasein expression of BDNF and NT3 in PCA was blocked by 50 μM Nrg treatment, while the effect of Nrg was abolished by addition of 10 μM Cyc. The above results confirmed that Nrg improved DA-induced memory impairment in MHE by activation of the Shh-neurotrophins signaling pathway.

## DISCUSSION

Currently, there is growing interest in the pathogenesis of MHE. We previously found that COMT inhibitor, a protein involved in accumulation of DA, was up-regulated in cirrhotic liver in MHE by 2-DE/MS; we then found increased levels of DA in cirrhotic liver and hippocampus in MHE rats [3]. Our present study further confirmed that elevated DA in the brain from cirrhotic liver could induce the memory impairment of MHE. We here demonstrated that chronic stimulation of cortical astrocytes significantly reduced the expression of proteins in the Shh pathway in MHE rats. Thus, we have provided the first evidence suggesting that the Shh pathway may be important in the pathogenesis of memory impairment in MHE rats. One study found that dopamine agonists impair or have no effect on stimulus-response learning and working memory [20]. Our study also indicated that increased dopamine impairs memory function. Furthermore, changes in DA levels *in vivo* and *in vitro* have been shown to regulate the expression of the Shh signaling cascade.

Blood-brain barrier disturbances are considered to be involved in the development of liver cirrhosis [21, 22] and in liver-brain signaling [23]. Hepatitis C virus also can cross the blood-brain barrier and affect cognitive ability [24]. Impaired permeability of the blood-brain barrier appears to be an important mechanism allowing DA into the brain during the development of MHE.

Recently, evidence has gathered suggesting that astrocytes are pivotal for learning and memory [25, 26]. Shh is expressed primarily in astrocytes in the cerebral cortex. In addition, the activity-dependent synthesis or secretion of Shh into the cerebral cortex by these astrocytes appears to be a mechanism for the regulation of neurogenesis [27]. Studies of Gli1 fate-mapping and postnatal knockdown of Smoin the postnatal forebrain raise the possibility that Shh is critical for signaling to GFAP-positive neural stem cells in the ventral subventricular zone [28], regulating discrete astrocyte populations, and maintaining astrocyte function [19, 29]. Our experiments showed a significant increase in expression of proteins in the Shh pathway in astrocytes of the cerebral cortex in MHE rats, suggesting that Shh signaling inactivation may be closely linked to the pathogenesis of MHE.

We examined whether Gli-mediated transcriptional regulation of genes related to memory was involved in memory impairment by DA in astrocytes. Neurotrophins (NTs), such as BDNF and NT-3, are secreted small proteins that are capable of signaling neurons to survive, differentiate, and grow. Although under physiological conditions, neurons produce neurotrophic factors, glial derived neurotrophins assume significance under neurodegenerative conditions, when neurons die and do not produce these invaluable factors. The loss of these neurotrophic factors has been implicated in the pathogenesis of various neurodegenerative diseases [32-34] and these neurotrophins have been shown to be required for synaptogenesis and synaptic function [35-37].

Our *in vitro* experiments revealed that Shh-induced neurotrophin expression was decreased by toxic levels of DA in MHE. Combined with these findings, we hypothesized that a substantial portion of the expression of endogenous neurotrophins, essential proteins for memory function, may rely on the Shh pathway in astrocytes of the cerebral cortex. During characterization of the pharmacological effect of DA, we found that neurotrophins levels in cerebral cortex showing memory impairment were substantially reduced. DA may be the major factor accelerating inactivation of signaling through the Shh pathway, thus inducing MHE. To examine how the function of neurotrophins is regulated by the Shh pathway, utilizing cyc, a Smo inhibitor, we found that transcriptional activation of neurotrophins could be attributed to transcription factor Gli; utilizing Shh, an activator of the Shh pathway, we found that DA desensitized IEG transcription to stimulation. During characterization of the Nrg-mediated memory-improving effect, we found that Nrg inversely sensitized neurotrophins transcription to stimulation by activating the Shh pathway in MHE models.

The cellular or molecular mechanism underlying these effects has not been fully established. The major findings of this study are as follows: DA inhibits memory function in MHE rats, and this effect requires inactivation of the Shh pathway because it could be partially stimulated by a selective agonist. Notably, the functional deterioration in the Shh pathway was reversed, concomitantly with memory improvement, by Nrg-mediated direct activation of the Shh pathway. Future experiments need to target the above aspects for an integrated description of the molecular mechanisms behind the effect of Nrg on memory regulation.

Nrg is a major flavonoid in grapefruit and gives grapefruit juice its bitter taste. It is metabolized to the flavanonenaringenin in humans. Both naringenin and hesperetin, which are the aglycones of Nrg and hesperidin, occur naturally in citrus fruits. In commercial grapefruit juice production, the enzyme Nrgase is used to remove the bitterness created by Nrg. These findings warrant a general recommendation to consume grapefruit juice regularly for disease prevention and support the idea that Nrg may have therapeutic uses in treating neurodegenerative disorders. Our findings not only provide a novel pathological hallmark in MHE but also a novel therapy for MHE.
